# Physicochemical and Antioxidative Characteristics of Potato Protein Isolate Hydrolysate

**DOI:** 10.3390/molecules25194450

**Published:** 2020-09-28

**Authors:** Chiung-Yueh Chang, Jinn-Der Jin, Hsiao-Li Chang, Ko-Chieh Huang, Yi-Fen Chiang, Shih-Min Hsia

**Affiliations:** 1 School of Nutrition and Health Sciences, College of Nutrition, Taipei Medical University, Taipei 110, Taiwan; d507104001@tmu.edu.tw (C.-Y.C.); a910241@gmail.com (K.-C.H.); yvonne840828@gmail.com (Y.-F.C.); 2 GeneFerm Biotechnology Co., Ltd., Tainan 741, Taiwan; jin168@geneferm.com (J.-D.J.); gracechang@geneferm.com (H.-L.C.); 3 Graduate Institute of Metabolism and Obesity Sciences, College of Nutrition, Taipei Medical University, Taipei 110, Taiwan; 4 School of Food and Safety, Taipei Medical University, Taipei 110, Taiwan; 5 Nutrition Research Center, Taipei Medical University Hospital, Taipei 110, Taiwan

**Keywords:** potato protein isolate, hydrolysate, physicochemical characteristics, solubility, antioxidant

## Abstract

This study investigated the physicochemical characteristics of potato protein isolate hydrolysate (PPIH) and its antioxidant activity. Potato protein isolate (PPI) was hydrolyzed into PPIH by the proteases bromelain, Neutrase, and Flavourzyme. Compared with PPI, the resulting PPIH had a lower molecular weight (MW, from 103.5 to 422.7 Da) and smaller particle size (<50 nm), as well as a higher solubility rate (>70%) under acidic conditions (pH 3–6). PPIH presented good solubility (73%) across the tested pH range of 3–6. As the pH was increased, the zeta potential of PPIH decreased from −7.4 to −21.6. Using the 2,2′-azino-bis-3-ethylbenzthiazoline-6-sulfonic acid (ABTS) radical-scavenging assay, we determined that the half-maximal effective concentration (EC_50_) values of ascorbic acid, PPIH, and PPI were 0.01, 0.89, and >2.33 mg/mL, respectively. Furthermore, PPIH (50 μg/mL) protected C2C12 cells from H_2_O_2_ oxidation significantly better than PPI (10.5% higher viability rate; *p* < 0.01). These findings demonstrated the possible use of PPIH as an antioxidant in medical applications.

## 1. Introduction

Potato is the world’s fourth most important food crop, and it contains abundant carbohydrates and proteins [1]. The potato starch industry releases large quantities of potato fruit juice (as a byproduct), which is rich in protein [2]. Potato protein has an excellent amino-acid score and is considered a nutritious dietary protein source [3]. Potato protein isolate (PPI) can be isolated from potato fruit juice using a combination of acidic precipitation and thermal coagulation [4]. The PPI is divided into three fractions: patatin (up to 40%), protease inhibitors (50%), and other high-molecular-weight (MW) proteins (10%). Patatin is a glycoprotein with an MW of 39–43 kDa (existing as native 80 kDa dimers) with different isoelectric point (*pI*) values ranging from pH 4.45–5.17. Patatin is a good emulsifier with foaming and gel-forming capacity and good stability [5]. Patatin contains several free-radical-scavenging amino acids, including tryptophan, phenylalanine, methionine, cysteine, histidine, and tyrosine, presenting antioxidant and angiotensin-converting enzyme, (ACE)-inhibitory activity [6,7]. Patatin has also been used to facilitate cheese ripening [8,9]. The protease inhibitors, with MWs ranging from 4.3 to 20.6 kDa, exhibit *pI* values of pH 5.1–9.0. The other high-MW proteins are mainly composed of oxidative enzymes including lipoxygenase, polyphenol oxidase, and several enzymes associated with starch synthesis [10].

Enzymatic hydrolysis is an attractive approach for utilizing proteins to achieve increased value [11,12]. Specifically, proteolysis is capable of breaking proteins into peptides with desired size, charge, and surface hydrophobic properties and further improving their antioxidative properties [13]. Enzymatic hydrolysis is capable of converting PPI into PPI hydrolysate (PPIH) with different molecular sizes, charges, and physicochemical characteristics. PPIH has attracted much attention in recent years due to its antioxidant activities. For example, PPIH showed antioxidative activity and reduced glutathione (GSH) produced by Fe(II)/H_2_O_2_-induced oxidation [10]. Kudo et al. [14] indicated that potato protein hydrolysate inhibited linoleic-acid oxidation and ferric-thiocyanate oxidation. Moreover, enzymatic hydrolysis has the favorable potential to remove antinutritive factors such as protease inhibitors [15]. Therefore, much attention has been paid to exploring the influences of enzymatic hydrolysis on the physicochemical and antioxidative properties of PPIH.

Endoproteases and exoproteases including bromelain, Neutrase, and Flavourzyme have been used to hydrolyze proteins to produce protein isolate hydrolysates showing antioxidant activities [16]. Among these proteases, bromelain (EC 3.4.22.32) is a cysteine endoprotease derived from pineapple stems, and it has also been demonstrated to be a safe and effective food supplement [17]. Neutrase (EC 3.4.24.28) is a neutral, zinc metalloendoprotease from *Bacillus amyloliquefaciens* with an optimum activity at pH 5.5–7.5 and 30–55 °C. This enzyme randomly hydrolyses internal peptide bonds to break down proteins to peptides. Furthermore, Flavourzyme is sold as a peptidase preparation from *Aspergillus oryza*e. This enzyme preparation is diversely and widely used for protein hydrolysis in research and industrial applications. In total, eight enzymes, namely, three endopeptidases (alkaline protease 1, EC 3.4.21.63; neutral protease 2, EC 3.4.24.39; neutral protease 1, EC 3.4.24), two aminopeptidases (leucine aminopeptidase 2, EC 3.4.11; leucine aminopeptidase A, EC 3.4.11), two dipeptidyl peptidases (dipeptidyl peptidase 4, EC 3.4.14.5; dipeptidyl peptidase 5, EC 3.4.14), and one amylase (α-amylase A type 3, EC 3.2.1.1), were identified [18]. These protein isolate hydrolysates are versatile and play a major role in a wide range of applications in the food industry.

Researchers have demonstrated the potent antioxidative activity of PPIH. They have also demonstrated its applicability as a food additive in vegetarian and elderly-friendly dishes and amino-acid supplements; however, the physicochemical characteristics of PPIH have not been reported. To investigate the physicochemical characteristics of PPIH and its antioxidant activity, PPI was hydrolyzed by bromelain, Neutrase, and Flavourzyme. The solubility, MW distribution, particle size, microstructure, and zeta potential of the resulting PPIH were determined. The 2,2′-azino-bis-3-ethylbenzthiazoline-6-sulfonic acid (ABTS) radical-scavenging assay of PPIH and its antioxidant effect on muscle C2C12 cells were also evaluated. Therefore, the aim of this study was to investigate the physicochemical characteristics of PPIH and its antioxidative activity on C2C12 murine myoblast cells.

## 2. Results and Discussion

### 2.1. Solubility of PPI and PPIH Samples under Acidic Conditions

**Figure 1:**
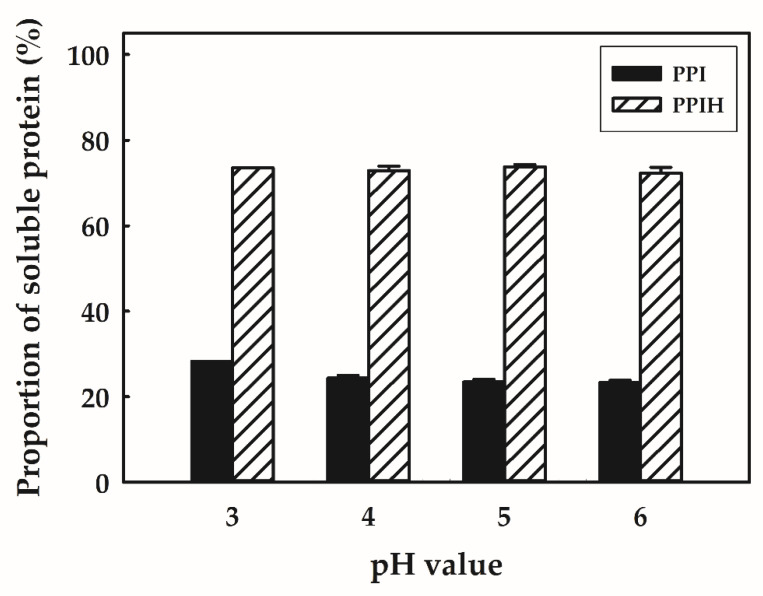
Effect of pH on the solubility of potato protein isolate (PPI) and PPI hydrolysate (PPIH). Each value is represented as the mean ±SD.

Protein solubility can also be affected by pH, and the *pI*s for most PPIs are between 4.5 and 6.5; therefore, proteins were precipitated in this pH region [2]. Figure 1 indicates the solubility of PPI and PPIH over a pH range of 3–6. PPI presented poor solubility across this pH range. A similar protein solubility profile of PPI was reported by Akbari et al. [11], with a solubility of ~30% at pH 3–5. The authors reported that PPI was prepared by heat coagulation and acid precipitation, and these processes cause PPI to have low solubility. Moreover, PPI generally aggregated at a pH level close to its isoelectric pH. By contrast, PPIH presented good solubility across the entire pH range. Compared to that of PPI, we noticed that the solubility of PPIH at its isoelectric pH was significantly increased. The solubility of PPIH was significantly higher than that of PPI in a pH range of 3–6. This result indicated that PPIH was more easily dissolved in acid solution than PPI. Hou et al. [19] indicated that the hydrolysis of proteins increased the functional properties of proteins such as solubility and emulsifying properties, mainly in heat-denatured proteins. Pęksa and Miedzianka [20] suggested that peptide bond cleavage and a decrease in the MW by enzymatic hydrolysis increased the number of ionizable carboxyl and amino groups (COO^−^ and NH_3_^+^, respectively). Therefore, the protein solubility improved due to the electrostatic repulsion between peptides and, thus, protein-water interactions. Therefore, the increased solubility of PPIH could be due to smaller peptides being produced by protease. As is known, a higher protein solubility is particularly important in acidic protein drinks in which sedimentation and precipitation are undesirable. Due to the increased PPIH solubility, especially at low pH, the application of these compounds will increase in acidic drinks and diets.

### 2.2. Zeta Potential of PPI and PPIH Samples under Acidic Conditions

**Figure 2:**
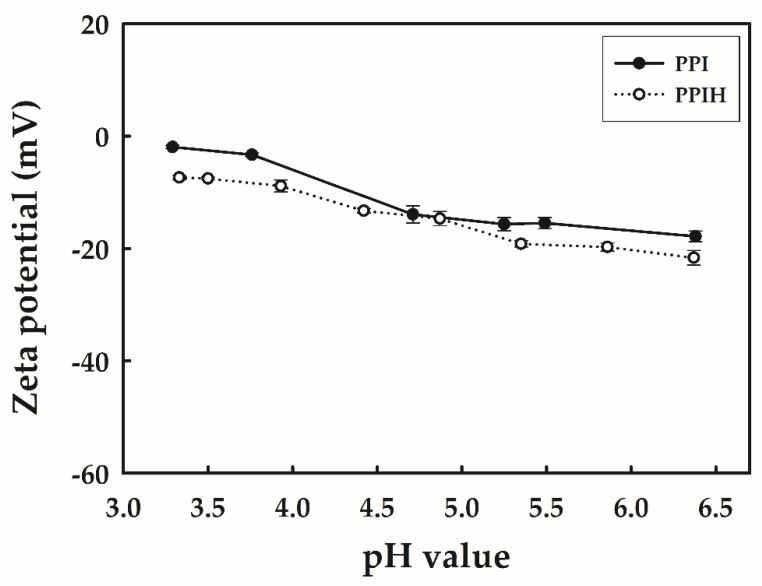
Effect of pH on the zeta potential of PPI and PPIH. Each value is represented as the mean ±SD.

Peptides are amphoteric molecules, containing both acidic and basic amino acids. The ionizable groups in proteins are the C-terminal carboxyl group, N-terminal amino group, and ionizable groups on side chains. Both α-carboxyl and α-amino groups are ionized, and the molecule becomes dipolar. Therefore, the zeta potentials of PPI and PPIH in a pH range of 3–6 were also investigated. As shown in Figure 2, as the pH was increased, the zeta potential of PPI decreased from −1.9 to −17.8. According to our results, the net negative charge of the PPI samples increased with increasing buffer pH. As mentioned previously, the *pIs* for PPI are between 4.5 and 6.5. The *pI* is the pH at which a particular molecule carries no net electrical charge. The net charge on the molecule is affected both by its own *pI* and by the pH of the buffer. At a pH above their *pI*, proteins carry a net negative charge; below their *pI*, they carry a net positive charge. This fact suggested that the zeta potential of PPI (1.9 ± 0.3 mV) at pH 3.3 was similar to the *pI*. Similar trends of zeta potential were also observed in the PPIH results. As the pH was increased, the zeta potential of PPIH decreased from −7.4 to −21.6. These results suggested that the zeta potentials of PPI and PPIH under acidic conditions ranged from 0 to −22 mV. Cheng et al. [21] reported that PPIH at pH 7.0 had a net negative charge, and the zeta potential of PPIH at pH 3–7 ranged from −0.2 to −6.2 mV. Schmidt et al. [22] also reported that the zeta potential of PPI at pH 7.0 was −5.7 mV. Chuacharoen and Sabliov [23] reported that the zeta potential has long been accepted as a good measure for assessing the stability of a nanoparticle system. The statistical analysis of the data revealed that there was a significant difference in zeta potential, whereas all values represented a good degree of stability, as it was reported that, with a zeta potential lower than −25 mV or higher than +25 mV, colloids could be obtained because of a greater electrostatic repulsion between nanoparticles [24].

### 2.3. Particle Size and TEM Analysis of PPI and PPIH Samples

**Figure 3:**
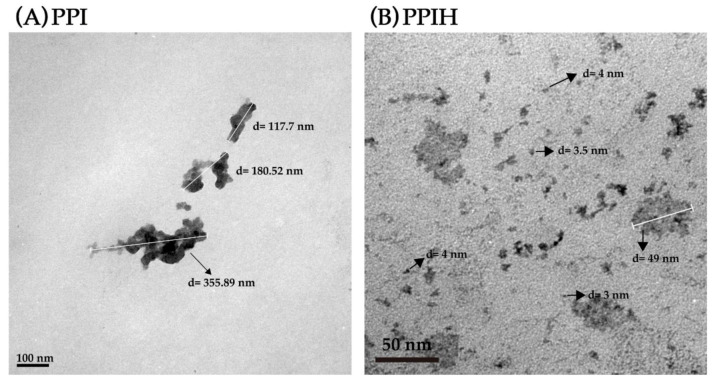
TEM analysis of PPI (**A**) and PPIH (**B**).

Klompong et al. [25] reported that enzymatic hydrolysis is used in the modification of protein structure, resulting in small peptides with improved physicochemical properties. Therefore, particle size and TEM analysis of PPI and PPIH were conducted. The particle size distribution of PPI was approximately as follows: 0–50 nm particles, 0% yield; 100–400 nm particles, 13% ± 4% yield; 7000–10,000 nm particles, 87 ± 4% yield. However, the particle size distribution of PPIH was approximately as follows: 0–50 nm particles, 100% yield; 100–400 nm particles, 0% yield; 7000–10,000 nm particles, 0% yield. We noticed that most of the particle sizes of the PPI and PPIH were approximately 7000–10,000 nm and 0–10 nm, respectively. This observation indicated that the particle size of PPIH was smaller than that of PPI. Midelfort and Wittrup [26] reported that proteins can form highly specific and structured complexes via self-assembly in a number of ways. These protein particles were formed as a result of aggregation and could span many orders of magnitude, from oligomers spanning tens of nanometers to visible aggregates spanning several hundred micrometers [27]. Ryan et al. [28] reported that TEM could be used to evaluate the aggregate shape of proteins. Therefore, the particle sizes of PPI and PPIH were also analyzed by TEM (Figure 3). As shown in Figure 3A, high-MW aggregates of PPI were observed. The length of these PPI particles was 117–355 nm. Furthermore, we found high-MW aggregates of PPIH, as shown in Figure 3B. The lengths of these PPIH particles were 3–50 nm. The microstructure of the PPI and PPIH displayed the appearance of aggregated nanoparticle structures. We noticed that compared with the PPI, the PPIH significantly decreased in typical dimensions. Therefore, the PPIH particles were smaller than the PPI particles. These TEM results suggested that PPI and PPIH were aggregated to form larger aggregates.

### 2.4. SDS-PAGE and Mass Spectrometry Analysis of PPI and PPIH

**Figure 4:**
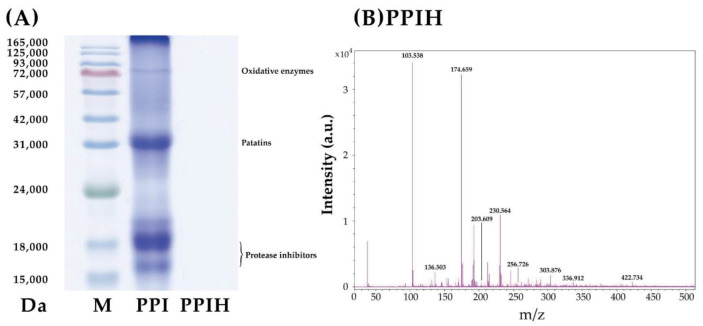
SDS-PAGE (**A**) and mass spectrometry analysis (**B**) of PPI and PPIH. M: MW standard.

The PPI can roughly be divided into patatin, protease inhibitors, and oxidative enzymes, which account for approximately 40%, 50%, and 10% of the total proteins, respectively [29]. As shown in Figure 4A, the PPI and PPIH samples were analyzed by SDS-PAGE, which separated patatin (~33 kDa), protease inhibitors (16–20 kDa), and oxidative enzymes (75–80 kDa) in the PPI sample. However, SDS-PAGE analysis indicated that no protein bands were observed in the PPIH sample. These results suggested the enzymatic hydrolysis of PPI to PPIH through peptide bond cleavage. Li et al. [30] suggested that protein hydrolysates have lower MW ranges than native proteins. Therefore, the PPIH sample was further analyzed by mass spectrometry (Figure 4B). The results indicated that the PPI was digested to hydrolysate with an MW of <500 Da, and most PPIH samples had mass-to-charge (*m*/*z*) ratios of 103.5–422.7. This observation indicated that smaller peptides were produced by enzymatic hydrolysis of PPI into PPIH.

### 2.5. Antioxidant Effects of PPIH on Muscle C2C12 Cells

Protein hydrolysates have been reported to have numerous health benefits such as antioxidative effects [31]. Li et al. [30] reported that hydrolysates with lower MW ranges had better antioxidant activity than native proteins. Therefore, the ABTS antioxidant activity of PPIH was evaluated. When 0.5, 0.75, 1.0, and 1.25 mg/mL PPIH were added, the ABTS radical-scavenging effect (%) values were 33.9% ± 1.4%, 44.4% ± 1.1%, 53.2% ± 1.0%, and 64.0% ± 0.8%, respectively. The PPIH sample presented antioxidant activity at various concentrations. The half-maximal effective concentration (EC_50_) values of ascorbic acid, PPIH, and PPI were 0.01, 0.89, and >2.33 mg/mL, respectively. The EC_50_ values of ascorbic acid, PPIH, and PPI were in the following order: ascorbic acid < PPIH < PPI. PPIH did not present the strong antioxidant properties of ascorbic acid and may, therefore, be suitable for medical purposes as an antioxidant. There is at present no evidence to support its use as a dietary supplement. Cheng et al. [32] reported that PPIH (5 mg/mL) exhibited strong ABTS radical-scavenging activity and antioxidant activity. Various peptides with specific amino-acid sequences are released after hydrolysis, and these protein hydrolysates possess significant antioxidant activity [33]. Kudo et al. [14] reported that the three peptides Phe-Gly-Glu-Arg, Phe-Gly-Glu-Arg-Arg, and Phe-Asp-Arg-Arg isolated from PPIH showed antioxidative activities. The antioxidative activities of these three peptides were compared to those of butylated hydroxyanisole. As a result, Phe-Gly-Glu-Arg, Phe-Gly-Glu-Arg-Arg, and Phe-Asp-Arg-Arg inhibited linoleic-acid oxidation by 55.3%, 61.7%, and 58.5% using the β-carotene decolorization assay system, respectively.

**Figure 5:**
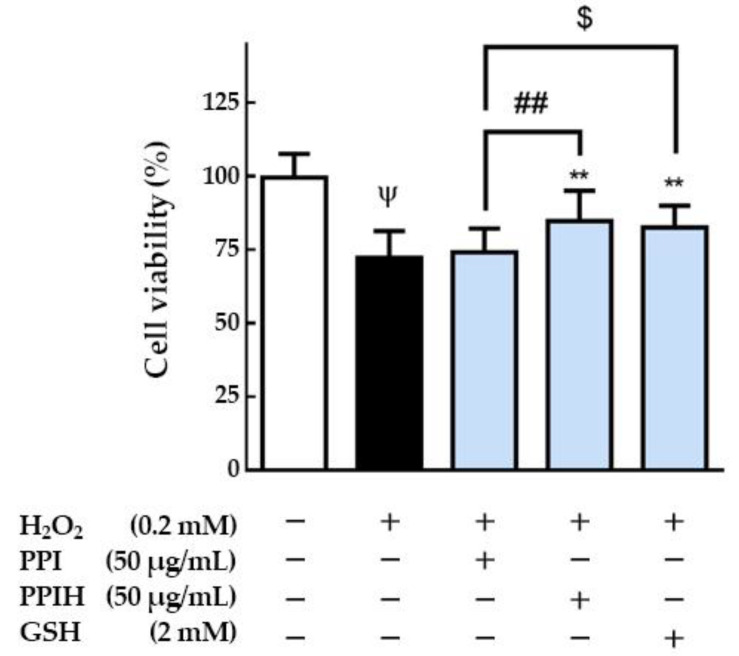
Effect of PPI and PPIH on the cell viability of C2C12 cells. C2C12 myoblasts were cultured (1 × 10^4^ cells) in 2% horse serum DMEM to differentiate for 6 days. After 6 days of differentiation, cells were pretreated with 50 μg/mL PPIH for 24 h and then treated with 0.2 mM H_2_O_2_ for 1 h. ^Ψ^ *p* < 0.05 versus control (white column), ^##^ *p* < 0.01 compared with PPI and PPIH, ^$^ *p* < 0.05 compared with PPI and GSH, ** *p* < 0.01 compared with the induced group (black column). PPIH: potato protein isolate hydrolysate. GSH: glutathione.

Furthermore, Hood et al. [34] reported that skeletal muscle has a unique ability to increase the use of oxygen during contraction. During intense activity, the high rate of O_2_ consumption in skeletal muscles can cause electron leakage from the electron transfer chain and incomplete oxygen reduction, leading to the generation of reactive oxygen species (ROS). Cellular and tissue injury is associated with ROS in many kinds of disorders [35]. Kerasioti et al. [36] reported that whey protein (0.5 mg/mL) showed protective activity against oxidative stress since a decrease in ROS levels in muscle cells (C2C12 cells) was observed. The whey protein was effective in scavenging H_2_O_2_ and protecting C2C12 cells from oxidative stress-induced damage. Thus, the protective effects of PPIH on H_2_O_2_-induced oxidative injury in skeletal muscle cells (C2C12 murine myoblast cells) were further evaluated. As shown in Figure 5, the results indicated that H_2_O_2_ (0.2 mM) or PPI (50 μg/mL) alone significantly reduced the cell viability of skeletal muscle cells compared with the control (without H_2_O_2_). Compared with PPI (50 μg/mL), PPIH (50 μg/mL) significantly increased the protective effect against H_2_O_2_-induced oxidative injury in skeletal muscle cells by 10.5% (*p* < 0.01).

**Figure 6:**
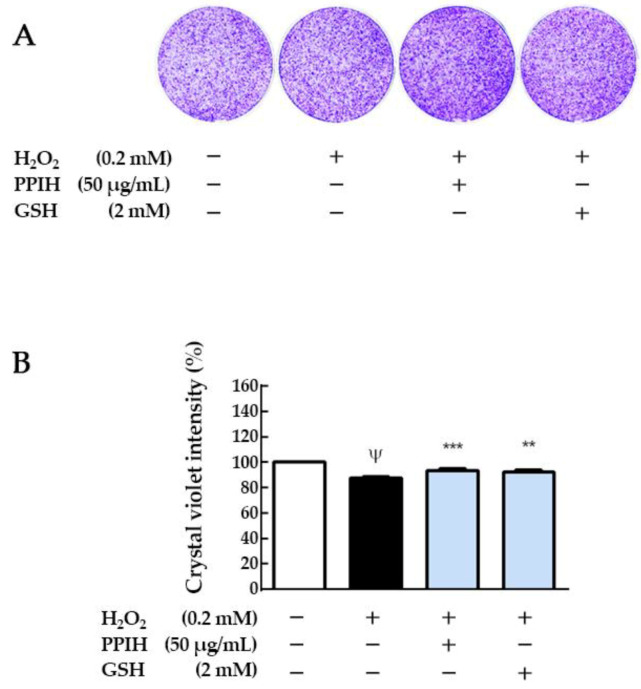
Effect of PPI and PPIH on the cell morphology of C2C12 cells. (**A**) Cell morphology. (**B**) Crystal violet intensity. C2C12 myoblasts were cultured (1 × 10^4^ cells) in 2% horse serum DMEM to differentiate for 6 days. After 6 days of differentiation, cells were pretreated with 50 μg/mL PPIH for 24 h and then treated with 0.2 mM H_2_O_2_ for 1 h. ^Ψ^ *p* < 0.05 versus control (white column), ** *p* < 0.01 or *** *p* < 0.001 compared with induced group (black column). PPIH: potato protein isolate hydrolysate. GSH: glutathione.

Moreover, GSH (2 mM) showed a protective effect against H_2_O_2_-induced oxidative injury (*p* < 0.01). The antioxidant GSH was used as a positive control in this study. GSH serves as a substrate for glutathione peroxidase to eliminate H_2_O_2_, and it exerts its antioxidant action by donating a hydrogen atom to a variety of radicals [36]. However, we also noticed that PPI (50 μg/mL) did not show a protective effect. Zhang et al. [37] reported that whey protein hydrolysates (200 µg/mL) reduced H_2_O_2_-induced apoptosis by 14% in a rat pheochromocytoma line 12. Our results suggested that PPIH showed a protective effect against H_2_O_2_-induced oxidative injury in skeletal muscle cells. Several studies in recent years have shown that protein hydrolysates from the enzymatic hydrolysis of plant proteins can act as a direct scavenger of various free radicals or as an antioxidant [38]. These results also indicated that PPIH could potentially be used as an antioxidant in medical applications. In addition, as shown in Figure 6A, the staining images showed that H_2_O_2_ induced cell morphology changes in C2C12 cells, and it can be found through image quantification that H_2_O_2_ reduced the number of C2C12 cells. Pretreatment with PPIH (50 μg/mL) had a protective effect (*p* < 0.001). GSH (2 mM) could also significantly restore the oxidative damage caused by H_2_O_2_ (Figure 6B). PPIH is a mixture of free-radical-scavenging amino acids and peptides from plants. It contains several amino acids (essential and nonessential), including branched-chain amino acids (BCAA; valine, leucine, and isoleucine) [39]. It also contains peptides (MW < 500 Da) presenting antioxidant activity, which were shown to protect C2C12 cells from H_2_O_2_ oxidation. Thus, PPIH could potentially be used as an antioxidant in medical applications and source of amino acid in vegetarian diets and elderly-friendly foods.

## 3. Materials and Methods

### 3.1. Preparation of PPI and PPIH

PPI was purchased from Roquette (Lestrem, France). PPIH was prepared by hydrolyzing PPI with the proteases bromelain (Chappion Biotechnology, Chiayi, Taiwan), Neutrase (Novozymes, Bagsvaerd, Denmark), and Flavourzyme (Novozymes, Bagsvaerd, Denmark). Briefly, PPI solution (100 mg PPI/mL) was prepared by dissolving PPI (1 kg) in distilled water (10 L) at 95 °C for a period of 1 h. The proteases bromelain (1000 CDU/mL), Neutrase (0.0024 AU-N/mL), and Flavourzyme (3.3 LAPU/mL) were then added to the PPI solution. The protease-containing PPI solution was incubated at 45 °C for 24 h. The hydrolyzed PPI solution was heated to 95 °C and maintained at that temperature for 1 h to halt protease activity. The PPI solution was then centrifuged at 9000× *g* at 4 °C for 10 min. Filtering the supernatant using No. 1 ADVANTEC filters resulted in a clear, yellow permeate (PPIH solution, 36 mg PPIH/mL). Note that the proteases (bromelain, Neutrase^®^, and Flavourzyme^®^) were denatured during the heating and filtration process and were, therefore, not included in the PPIH. Finally, the PPIH solution was freeze-dried and held in an airtight container at 25 °C prior to use.

### 3.2. Solubility of PPI and PPIH under Acidic Conditions

PPI and PPIH samples (100 mg) were resuspended in 10 mL of citrate-phosphate buffer (0.1 M, pH 3–6). The samples were stirred at 25 °C for 10 min and then centrifuged at 12,000× *g* for 20 min at 25 °C. The protein content of PPI in the precipitate and protein content of PPIH in the supernatant were determined by the Kjeldahl method according to Shen and Kuo [40]. The protein solubility was estimated as the percentage ratio of the protein content of the supernatant to the total original sample’s protein content. The protein solubility determinations of all samples were conducted in triplicate. The solubilities of PPI and PPIH were calculated as follows:

(1) $$\mathrm{PPI}\;\mathrm{solubility}\;\left(\%\right)=100\;-\left(\frac{\mathrm{protein}\;\mathrm{content}\;\mathrm{of}\;\mathrm{precipitate}}{100\;\mathrm{mg}}\right)\times \;100$$

(2) $$\mathrm{PPIH}\;\mathrm{solubility}\;\left(\%\right)=\;\frac{\mathrm{protein}\;\mathrm{content}\;\mathrm{of}\;\mathrm{supernatant}}{100\;\mathrm{mg}}\times \;100$$

### 3.3. Determination of the MW Distributions of PPI and PPIH

The MW distributions of PPI and PPIH were determined by sodium dodecyl sulfate polyacrylamide gel electrophoresis (SDS-PAGE) according to Laemmli [41]. SDS-PAGE analysis of PPI and PPIH was performed with a 12.5% separation gel and 5% stacking gel. PPI (6 mg) and PPIH (6 mg) were separately mixed with 1 mL of buffer (0.02% bromophenol blue, 2% SDS, 10% glycerol, 5% β-mercaptoethanol, and 70 mM Tris-HCl, pH 6.8). Next, the samples were heated at 95 °C for 7 min. Samples (10 μL) and protein ladders (6 μL) were loaded into separate wells. After electrophoresis, the gels were stained with Coomassie Brilliant Blue R-250. After staining, the gel was washed with 10% acetic acid to destain. The stained gel was digitized using an image scanner (Epson America Inc., Long Beach, CA, USA). PPIH was also analyzed by an Autoflex III mass spectrometer (Bruker Daltonik, Bremen, Germany). The masses in the range of 0–500 were measured. Each sample was analyzed in triplicate.

### 3.4. The Particle Size of PPI and PPIH

Particle size distributions (PSDs) of PPI and PPIH were obtained on the basis of the method of Win and Feng [42] using a 90Plus Nanoparticle Size Analyzer (Brookhaven Instruments, Holtsville, New York, NY, USA). PPI and PPIH solutions (0.05% *w*/*v*) were prepared using the required number of samples solubilized in dd water and stirred for 20 min at 25 °C. The PPI and PPIH solutions were centrifuged at 12,000× *g* for 20 min at 25 °C. The supernatants of the PPI and PPIH solutions (3 mL) were loaded into a cuvette to measure particle size. By using the principles of dynamic light scattering, the PSD was obtained from the velocity distribution of particles suspended in a dispersing medium. The signal was analyzed with sizes ranging from 1 nm to 10 µm. Each sample was analyzed in triplicate.

### 3.5. Microstructure of PPI and PPIH

The microstructures of PPI and PPIH were determined by transmission electron microscopy (TEM) according to Wang et al. [43]. The TEM images of PPI and PPIH were determined using a JEM-1400 instrument (JEOL Co. Ltd., Tokyo, Japan) operated at 100 keV. A 300-mesh formvar carbon-coated copper grid was used as the support substrate. The sample was prepared by dropping 10 μL of PPI and PPIH solutions onto the grids and blotting the excess sample after 3 min. Positive staining was obtained by adding uranyl acetate to the sample solutions on the TEM grids. Excess uranyl acetate was blotted and then allowed to air-dry. By using Lispixl (NIST freeware), the statistics describing the size distribution of the observed colloids were analyzed. Each sample was analyzed in triplicate.

### 3.6. Zeta Potential Measurements

The zeta potential measurements of PPI and PPIH as a function of pH were performed on the basis of the method of Tang and Sun [44] using a Zetasizer Nano ZS (Malvern Instruments, Malvern, Worcestershire, UK). Samples were prepared at 1% under acidic conditions (pH 3–6) adjusted with HCl/NaOH solutions. The samples were stirred at 20 °C before analysis and then centrifuged at 10,000× *g* for 10 min. The samples were then measured in triplicate at 20 °C. By using the Henry equation and the Smoluchowski approximation, the zeta potential was calculated from the electrophoretic mobility. Each sample was analyzed in triplicate.

### 3.7. ABTS Radical-Scavenging Assay

The ABTS radical-scavenging assay was determined on the basis of the method of Re et al. [45]. The ABTS solution was prepared by reaction of 5 mL of a 7 mM ABTS solution and 89 μL of a 140 mM potassium persulfate solution. The working solution was further diluted in water until the initial absorbance value of 0.7 at 734 nm. The PPIH sample (160 μL) was mixed with 40 μL of ABTS solution and mixed with ABTS solution in a 96-well microplate at 30 °C for 5 min. The absorbance was measured by a microplate reader (Molecular Devices Corporation, Sunnyvale, CA, USA) at 734 nm. The half-maximal effective concentration (EC_50_) of the ABTS free-radical-scavenging activity was calculated from the linear regression curve of percentage inhibition against concentration and further used to determine the antioxidant activity. All determinations were performed in triplicate. The absorbance values were corrected for radical decay using blank solutions.

(3) $$\mathrm{ABTS}\;\mathrm{radical}\;\mathrm{scavenging}\;\mathrm{activity}\left(\%\right)=\left(1\;-\;\frac{\mathrm{Abs}734\;\mathrm{of}\;\mathrm{sample}}{\mathrm{Abs}734\;\mathrm{of}\;\mathrm{control}}\right)\times \;100$$

### 3.8. Cell Culture

The C2C12 murine myoblast cell line (BCRC 60083), obtained from the Culture Collection and Research Center (CCRC) at the Food Industry Research and Development Institute (Hsinchu, Taiwan), was cultured in Dulbecco’s modified Eagle’s medium (DMEM, Gibco, Thermo Fisher Scientific, Inc., Waltham, MA, USA) with 10% fetal bovine serum at 37 °C with 5% CO_2_. To induce differentiation, 70–80% confluent cells were cultured in a differentiating medium consisting of DMEM supplemented with 2% horse serum, which was refreshed every two days. After 6 days of differentiation, multinuclear myotubes were formed.

### 3.9. Cell Viability Assay

To evaluate the protective effects of PPI and PPIH on H_2_O_2_-induced oxidative injury in skeletal muscle cells, cell viability was determined using a colorimetric 3-(4,5-dimethylthiazol-2-yl)-2,5-diphenyltetrazolium bromide (MTT) assay according to the method of Bahuguna et al. [46]. C2C12 cells were seeded in 96-well plates at a density of 0.5–1.0 × 10^4^ cells/well and pretreated with various concentrations of PPI (50 μg/mL), PPIH (50 μg/mL), and GSH (2 mM) in serum-free medium for 24 h. Cells were further treated with 0.2 mM H_2_O_2_ for 1 h, after which cell viability was determined by MTT assay. Subsequently, the MTT solution (1 mg/mL) was added directly to each well (100 μL/well) for 2 h. The cells were then dissolved in DMSO (100 μL/well). The absorbance was measured on an Epoch Microplate Spectrophotometer (BioTek, VT, USA) at 570 nm, with a reference wavelength at 630 nm. C2C12 cells were also seeded in six-well plates at a density of 1 × 10^5^ cells/well and then stained with crystal violet dye. The color intensity quantification was evaluated by ImageJ software (version 1.52, National Institute of Health, Bethesda, MD, USA). Each sample was analyzed in triplicate.

### 3.10. Statistical Analysis

Data are expressed as the mean ±standard deviation (SD). Student’s *t*-test was used for comparisons between two groups. The statistically significant differences between treatments were determined by one-way ANOVA, and the significance level was set at *p* < 0.05. All analyses were performed using GraphPad Prism software version 6.0 for Windows (GraphPad Software, San Diego, CA, USA).

## 4. Conclusions

Our results identified patatin, protease inhibitors, and oxidative enzymes as major proteins in PPI. These proteins were hydrolyzed to PPIH by bromelain, neutral enzymes, and flavor enzymes, resulting in small peptides with improved physicochemical and antioxidative properties. The MW of PPIH (<500 Da) was lower than that of PPI, the particle size was smaller (<50 nm), and solubility was higher (>72%). These properties make PPIH an ideal additive for a wide range of foods, such as drinks and jellies. PPIH also demonstrated ABTS radical-scavenging activity capable of protecting C2C12 cells from H_2_O_2_ oxidation. Overall, our results indicate that PPIH could be used as an antioxidant in medical applications.
